# The influence of stimuli valence, extraversion, and emotion regulation on visual search within real-world scenes

**DOI:** 10.1038/s41598-022-04964-y

**Published:** 2022-01-18

**Authors:** Robert C. A. Bendall, Peter Eachus, Catherine Thompson

**Affiliations:** Directorate of Psychology and Sport, School of Health and Society, University of Salford, Allerton Building, Frederick Road, Salford, M5 4WT UK

**Keywords:** Psychology, Human behaviour, Emotion

## Abstract

Affective traits, including extraversion and emotion regulation, are important considerations in clinical psychology due to their associations with the occurrence of affective disorders. Previously, emotional real-world scenes have been shown to influence visual search. However, it is currently unknown whether extraversion and emotion regulation can influence visual search towards neutral targets embedded within real-world scenes, or whether these traits can impact the effect of emotional stimuli on visual search. An opportunity sample of healthy individuals had trait levels of extraversion and emotion regulation recorded before completing a visual search task. Participants more accurately identified search targets in neutral images compared to positive images, whilst response times were slower in negative images. Importantly, individuals with higher trait levels of expressive suppression displayed faster identification of search targets regardless of the emotional valence of the stimuli. Extraversion and cognitive reappraisal did not influence visual search. These findings add to our understanding regarding the influence of extraversion, cognitive reappraisal, and expressive suppression on our ability to allocate attention during visual search when viewing real-world scenes.

## Introduction

Theories suggest that attentional resources are biased towards task-relevant stimuli (top-down processing)^1^ and to salient stimuli (bottom-up processing)^2^. However, it has been proposed that these distinctions are unable to explain all attentional processing^3^. For instance, it has been argued that the emotional valence of stimuli may provide an additional type of influence on attentional processing and selective attention^4–6^. This influence has been termed emotional attention^7^ and research exists demonstrating that emotional processing, top-down processing, and bottom-up processing can exert separate influences on selective attention^4,8^.

Studies have shown that emotional targets are detected more accurately and faster in a range of tasks, including during visual search^9,10^. Additionally, emotional distractors capture attention more than neutral distractors^6^. Most research studying the influence of image valence on attention uses faces or non-naturalistic arrays containing targets and/or distracters. Fewer studies have used naturalistic visual scenes, although those that do show similar effects of valence. For instance, it was shown that when participants were required to decide how many humans were located within a visual scene, response times were slower when the scene was negative compared to neutral^11^. Further, it has been shown that the emotional content of real-world scenes also influences fixation number and duration whereby individuals make more fixations on negative and positive stimuli compared to neutral^12^, whilst the duration of fixations has been shown to vary in emotional and neutral scenes^12–14^. Recently, contextual cueing has also been shown to be influenced by task-irrelevant emotional stimuli. Contextual cueing studies include the repeated presentation of specific target-distractor arrangements and have suggested that the learned association between target and distractor can influence visual search. The observation that contextual cueing is influenced by emotional stimuli suggests that emotional stimuli can impact the encoding of spatial target-distractor associations, and by extension, impact visual context memory for the environment^15^.

Bendall et al.^16^ investigated visual search within emotional and neutral real-world scenes asking participants to search for neutral target letters embedded within positive, negative, and neutral scenes. Image valence had an impact on target search, with lower accuracy when the target was embedded in positive images and slower response times when the target was embedded in negative images. Together with the earlier findings this shows that emotional valence of stimuli therefore has an impact on visual attention, adding to the influence of bottom-up and top-down processing.

There is also an argument that personality and individual differences can influence visual attention. Related to this, some personality traits and individual differences are linked to affective reactivity—conceptualised here as individual responses to stressors and/or emotional stimuli. For instance, one trait linked to affective reactivity which features in models of psychopathology is extraversion^17,18^, and it is associated with positive emotions, higher wellbeing, and more positive experiences^19^. Models of depression link low levels of extraversion to Major Depressive Disorder (MDD)^20^ and depressed individuals show reduced reactivity to emotional stimuli^21,22^. Additionally, research evidencing emotion-congruent attentional biases in affective disorders demonstrates the links between attentional and emotional processes in psychopathology, highlighting attention as a key mechanism in the development of such disorders^23^. Further support for this comes from findings that show content-sensitive attentional biases and attentional biases towards emotional faces are evident in individuals with post-traumatic stress disorder^24^.

Extraversion has been found to affect processing of neutral stimuli. For example, using a rapid serial visual presentation task, MacLean and Arnell^25^ found that greater extraversion predicted a smaller attentional blink (AB). Early theories to account for the AB effect suggest that it occurs due to a limited processing capacity whereby individuals are unable to process information presented in close temporal proximity^26^. These results would therefore indicate that extraverts can process more information and consequently suffer from the AB to a lesser extent. However, other theoretical work proposes that the AB is not due to attentional capacity limits. Instead, it has been suggested that a gating system or attentional filter helps to prioritise relevant information and suppress irrelevant information^27^. In the AB task it is proposed that the distractor presented after the target receives a processing boost resulting in an inhibitory feedback response. According to this perspective it would be argued that extraverts have a more effective attentional filter and are better at inhibiting distractor items compared to introverts. Extraversion has also been found to predict attentional performance during change detection where improved accuracy is associated with higher levels of extraversion, suggesting that extraverts allocate attention more effectively in demanding tasks^28^.

It has also been theorised that extraversion influences attentional bias to emotionally valanced stimuli^29–31^. For example, extraversion increases attention towards positive information and reduces attention towards negative information^32^. Additionally, extraversion is related to increased neural responses to positive stimuli compared to neutral stimuli and is linked to sustained attention to positive stimuli^33–35^. Therefore, there is evidence suggesting that extraversion is associated with emotional attention at both the behavioural and neural level.

A further individual difference trait linked to affective reactivity is emotion regulation (ER), broadly defined as the initiation of a conscious or non-conscious effort to start, stop, or modulate an emotion^36^. Poor ER is related to affective disorders such as depression and anxiety^37^, and individual differences in habitual ER strategy use may determine susceptibility and resilience when presented with affective stressors. The most extensively studied strategies are cognitive reappraisal; an antecedent-focussed strategy where individuals reappraise a potentially emotional situation in a way that changes its emotional impact, and expressive suppression; a response-focused strategy where individuals inhibit ongoing emotion-expressive behaviour^38^. Cognitive reappraisal is related to reduced negative affect^39,40^, whilst expressive suppression has been associated with both an increase^41–43^ and a decrease^44^ in negative affect.

Viviani (2013) has argued that the prevailing theoretical view of ER encompasses a dual-process model^45,46^ involving both the automatic encoding of emotional stimuli and the integration of prefrontal cognitive control mechanisms^47–49^. This standpoint is in line with models of attention whereby bottom-up processing (similar to automatic encoding of emotional stimuli) and top-down processing (similar to cognitive control mechanisms) guide the allocation of attentional resources^1,2^. Additionally, there is overlap between brain structures that underpin ER, attention, and top-down cognitive control^50–54^ suggesting that attentional control and ER share common mechanisms.

Studies have shown that ER can influence visual attention. For example, research has demonstrated that regulating negative affect reduces dwell time to arousing areas of unpleasant images compared to when passively viewing unpleasant images^55–57^. Additionally, Strauss et al.^58^ have shown that successful down regulation of negative affect is associated with differing patterns of eye movements across ER strategies. For instance, when participants were required to adopt reappraisal, initial increases in dwell time to arousing scene regions were followed by a move away from the same scene regions later in the trial. In contrast, when adopting suppression, participants displayed reduced dwell time to arousing areas during the complete trial. Moreover, when regulating emotion, variations in fixations have been shown to account for 35–78% of the variance in brain activity, demonstrating that gaze fixations are predictive of changes in brain activity during an ER task^57^.

Research therefore suggests that extraversion and instructed ER can influence the allocation of attention to both neutral and emotional information. However, it is currently unclear whether trait levels of extraversion and habitual use of ER strategies can influence visual search during tasks involving emotional stimuli and neutral targets. Previous research investigating the links between ER and attention have employed tasks where participants are explicitly instructed to adopt ER strategies, and consequently it is not presently understood whether habitual use of ER strategies impacts attention and visual search when there is no explicit instruction to adopt a specific regulation strategy.

The aim of the current study was to investigate whether the influence of stimuli valence on the allocation of attention varies due to extraversion and habitual ER strategy use. The visual search task used by Bendall et al.^16^ was adopted with the additional inclusion of measures of extraversion and habitual ER strategy use. Participants were required to identify neutral targets embedded within images varying in emotional valence (positive, neutral, or negative). Following the results of Bendall et al.^16^ it was predicted that accuracy would be reduced for positive trials and that reaction time would be longer for negative trials. Additionally, it was predicted that individuals reporting greater levels of extraversion, cognitive reappraisal, and expressive suppression would show improved behavioural task performance compared to individuals reporting low levels of these traits (i.e., faster response times and fewer errors). Although expressive suppression is often associated with increased negative affect^37,41–43^, it is proposed that increased levels of expressive suppression would benefit the individual when completing the visual search task. Within a regulation context, expressive suppression involves the inhibition or suppression of ongoing emotion-expressive behaviour. In the current task participants were required to search for a target whilst ignoring or suppressing the real-world scenes. Therefore, it was predicted that individuals who more frequently adopt expressive suppression in daily life would be more able to inhibit the real-world scenes, resulting in more accurate and/or quicker target identification. Importantly, it was also predicted that these individual difference traits would show an interactive effect with stimuli valence; performance of individuals with greater levels of extraversion, cognitive reappraisal, and expressive suppression (but not individuals with lower levels of these traits) would show improved performance during the positive and negative trials compared to the neutral trials.

## Results

Data collected included accuracy (percentage correct; %) and response times (seconds; s). 96.48% of trials were completed accurately and participants took an average of 2.00 s to correctly identify the target. 136 trials were terminated (0.01% of trials), and 116 trials (2.48% of total correct trials) were removed at ± 2 standard deviations from the mean for reaction time. Outliers were removed at ± 2 standard deviations from the mean resulting in two participants being removed based on accuracy, and a further participant was excluded based on reaction time.

Linear mixed-effects models were used to assess the influence of stimuli valence, cognitive reappraisal, expressive suppression, and extraversion on visual search (accuracy and response time). Initially, relevant regression assumptions were checked. The assumptions of normally distributed errors and homogeneity of variance and linearity were shown to be met after visual inspection of the histograms of standardised residuals, scatterplots of standardised residuals, and normal P-P plots of standardised residuals. Additionally, no outliers were present as indicated by an analysis of standardised residuals (standardised residual minimum =  − 1.871, standardised residual maximum = 2.396). The assumption of independent errors was met indicated by a Durbin-Watson value of 1.819. The assumption of non-zero variances was also met (all scores > 1.074). Importantly, no collinearity was present within the data as indicated by all tolerance scores > 0.724 and all variance inflation factors < 1.380. Within the linear mixed-effects models, stimuli valence, cognitive reappraisal, expressive suppression, and extraversion were set as fixed effects.

**Figure 1:**
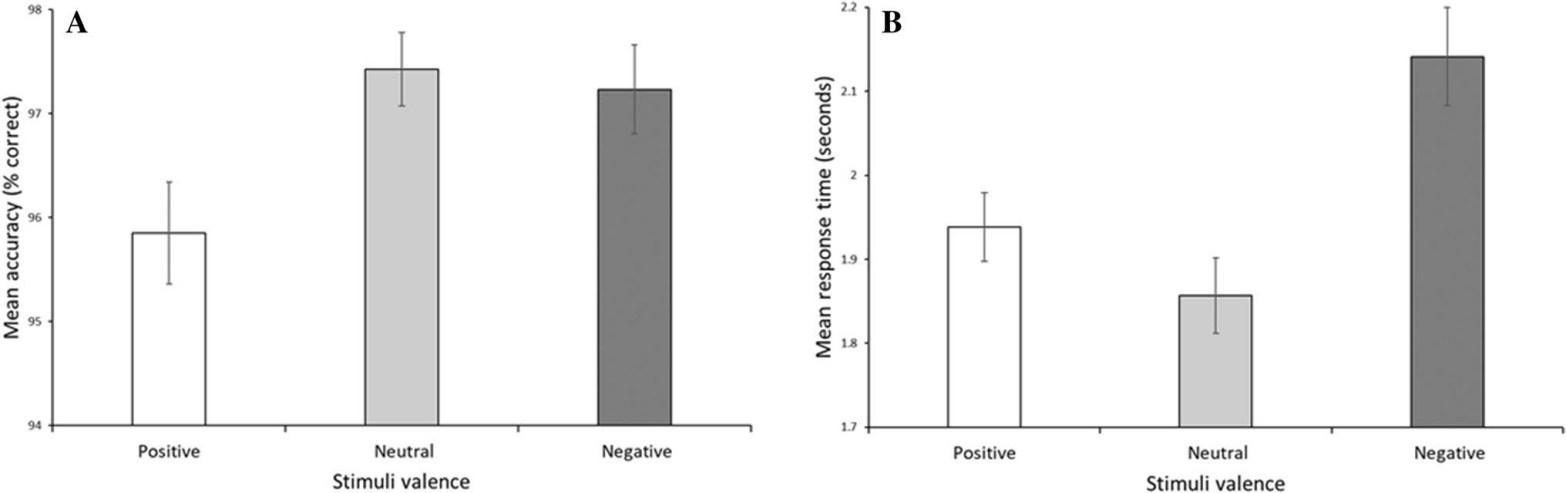
Mean accuracy (percentage correct) in the visual search task (**A**). Neutral trials were completed more accurately than positive trials. Error bars = standard error of the mean. Mean response time (seconds) in the visual search task (**B**). Neutral trials were completed quicker than positive and negative trials. Error bars = standard error of the mean.

For accuracy, there was a significant main effect of stimuli valence, *F*(2, 102.895) = 3.539, *p* = 0.033; Fig. 1A. Planned comparisons show that targets embedded within neutral scenes (*M* = 97.43, *SD* = 2.45) were identified more accurately than targets embedded within positive scenes (*M* = 95.85, *SD* = 3.40), β =  − 1.574, *SE* = 0.611, *p* = 0.012, 95% CI =  − 2.790, − 0.359. There were no differences in accuracy between neutral scenes (*M* = 97.43, *SD* = 2.45) and negative scenes (*M* = 97.23, *SD* = 2.97), β =  − 0.192, *SE* = 0.561, *p* = 0.734, 95% CI =  − 1.306, 0.923. Additional post-hoc analysis (corrected for multiple comparisons; α = 0.125) show that targets embedded within negative scenes (*M* = 97.23, *SD* = 2.97) were identified more accurately than targets embedded within positive scenes (*M* = 95.85, *SD* = 3.40), *t*(46) = 3.260, *p* = 0.002, *d* = 0.474, CI = 0.529, 2.237. The main effect of extraversion was non-significant, *F*(1, 127.326) = 0.268, *p* = 0.605; β = 0.016, *SE* = 0.031, 95% CI =  − 0.045, 0.078. The main effect of expressive suppression was non-significant, *F*(1, 127.117) = 0.001, *p* = 0.979; β = 0.005, *SE* = 0.213, 95% CI =  − 0.415, 0.426. The main effect of cognitive reappraisal was non-significant, *F*(1, 127.625) = 0.321, *p* = 0.572; β =  − 0.135, *SE* = 0.239, 95% CI =  − 0.609, 0.338. The interactions between stimuli valence and affective traits were all non-significant; all *F*s < 0.2.418, all *p*s > 0.079.

**Figure 2:**
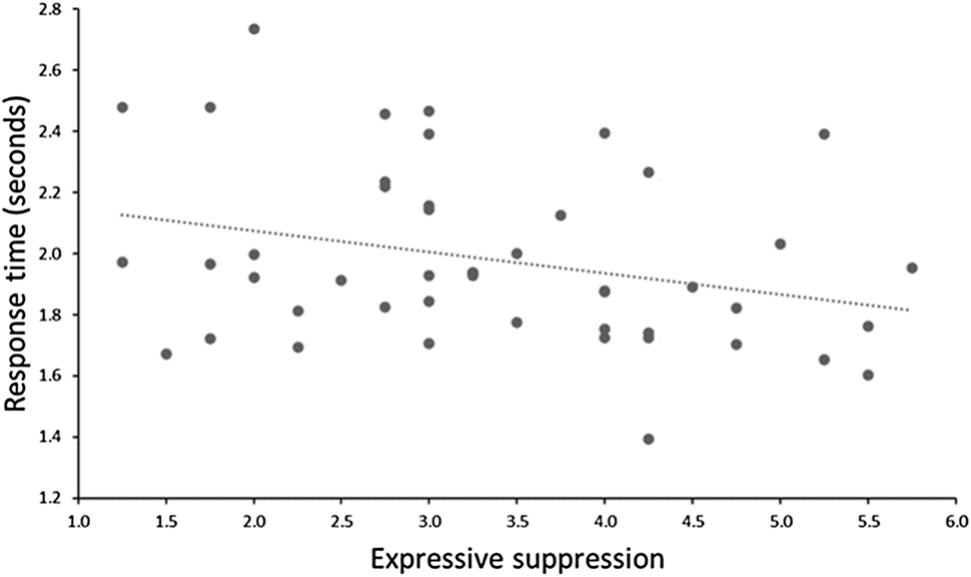
Individuals reporting higher levels of habitual use of expressive suppression completed visual search trials quicker than individuals reporting lower habitual use of expressive suppression.

For response time, there was a significant main effect of stimuli valence, *F*(2, 102.308) = 7.389, *p* < 0.001; Fig. 1B. Planned comparisons show that targets embedded within neutral scenes (*M* = 1.86, *SD* = 0.31) were identified faster than targets embedded within negative scenes (*M* = 2.14, *SD* = 0.40), β = 0.285, *SE* = 0.075, *p* < 0.001, 95% CI = 0.137, 0.433. There were no differences in response time between neutral scenes (*M* = 1.86, *SD* = 0.31) and positive scenes (*M* = 1.94, *SD* = 0.28), β = 0.083, *SE* = 0.062, *p* = 0.183, 95% CI =  − 0.040, 0.205. Additional post-hoc analysis (corrected for multiple comparisons; α = 0.125) show that targets embedded within positive scenes (*M* = 1.94, *SD* = 0.28) were identified faster than targets embedded within negative scenes (*M* = 2.14, *SD* = 0.40), *t*(46) =  − 4.086, *p* < 0.001, *d* = 0.598, CI =  − 0.302, − 0.103. The main effect of extraversion was non-significant, *F*(1, 123.210) = 0.169, *p* = 0.682; β = 0.001, *SE* = 0.004, 95% CI =  − 0.006, 0.009. However, the main effect of expressive suppression was significant, *F*(1, 121.646) = 7.847, *p* = 0.006; β =  − 0.066, *SE* = 0.024, 95% CI =  − 0.113, − 0.019; Fig. 2. Participants reporting more frequent habitual use of expressive suppression were quicker to correctly identify visual search targets. The main effect of cognitive reappraisal was non-significant, *F*(1, 123.387) < 0.001, *p* = 0.990; β = 0.001, *SE* = 0.027, 95% CI =  − 0.054, 0.055. The interactions between stimuli valence and affective traits were all non-significant; all *F*s < 2.724, all *p*s > 0.055.

## Discussion

The current study was designed to investigate whether the influence of stimuli valence on visual attention varies according to extraversion and trait levels of habitual ER strategy use. Participants completed questionnaires recording levels of extraversion and habitual use of two ER strategies; cognitive reappraisal and expressive suppression, followed by a visual search task where targets were presented within real-world scenes of varying emotional valence; positive, neutral, or negative.

As predicted, participants were less accurate at identifying targets presented within positive real-world scenes compared to neutral real-world scenes and negative real-world scenes. Participants correctly identified the search target equally well when embedded within neutral and negative scenes. Previously, using the same visual search task, participants were more accurate at identifying targets within neutral real-world scenes compared to positive (but not negative) real-world scenes^16^. For response time, participants were slower to correctly identify a target when it was presented on a negative image compared to a neutral image, supporting our hypothesis and previous findings^16^. Also in line with our predictions, there was no difference in response time to correctly identify a target when embedded within neutral and positive scenes. Additionally, participants were slower to correctly identify a target when it was embedded within a negative real-world scene compared to a positive real-world scene. Taken together, these results suggest that attention varies according to the emotional valence of real-world scenes and provide a successful replication of earlier findings^16^. Moreover, the differing influences of positive and negative stimuli on reaction time and accuracy suggest that emotional stimuli may impact attentional processing style in different ways. This suggestion is supported by recent findings suggesting that positive stimuli can impair contextual cueing whereas negative stimuli can enhance contextual cueing^15^. The current findings suggest that positive real-world scenes impair visual search accuracy illustrated by the impaired identification of targets. Since this came with no impact on response time to locate a target it suggests effective localisation, but poor identification. In contrast, the effects of negative real-world scenes with longer response times and no impact on accuracy are suggestive of impaired localisation of the search target without any impact on target identification (see Bendall et al.^16^ for further discussion). Previous research has suggested that positive emotion (rather than the emotional valence of stimuli) can broaden visuospatial attention^59,60^ (however see^61,62^). This could be investigated further by testing whether the effects of emotion and stimuli valence have separate, interactive, or overlapping influences on visual search.

Importantly, habitual use of expressive suppression was shown to influence the speed to correctly identify a target. Individuals reporting more frequent habitual use of expressive suppression correctly identified search targets quicker than individuals reporting less frequent habitual use of expressive suppression. This novel finding, supporting our a priori hypothesis, suggests that increased habitual use of expressive suppression provides a benefit during visual search regardless of the emotional valence of the scenes that targets are presented within. It is important to note that whilst expressive suppression is often referred to as a maladaptive ER strategy, due to its association with increased negative affect^37,41–43^, it is uniquely suited to confer benefits in the completion of the visual search task adopted in this study. Expressive suppression, within a regulation context, involves the inhibition or suppression of ongoing emotion-expressive behaviour. The current task required individuals to respond to a search target whilst ignoring or suppressing the real-world scenes that were presented simultaneously. Therefore, individuals who more frequently adopt expressive suppression as an ER strategy are likely to be skilled at suppressing information in daily life. Our findings demonstrating faster response times in individuals who more often adopt expressive suppression suggests that this ER strategy may be linked to improved cognitive functioning, including visual attention. This argument is supported by previous research showing that successful ER is related to improved behavioural performance and neural activation during completion of a Stroop task^63^. Together with the findings reported here, these studies suggest that expressive suppression is associated with improved executive functioning (e.g., cognitive control/response inhibition and visual search). One possible mechanism linking ER with improved visual search performance is visual working memory ability and this is an area for further investigation. In support, it has recently been shown that working memory training improves ER ability^64,65^.

Habitual use of cognitive reappraisal did not impact accuracy or response time in the visual search task. One strength of the current study is that it adopted a more naturalistic approach measuring habitual use of ER strategies rather than instructing participants to adopt specific ER techniques during task completion. A similar approach has helped to develop our understanding of the neurobiological mechanisms supporting ER^66,67^. The findings from the current study suggest that habitual use of expressive suppression, but not cognitive reappraisal, is associated with reduced response time during a visual search task. Further work investigating additional ER strategies may help to elucidate if this benefit is specific to expressive suppression and/or exclusive to the specific visual search task adopted in the current work.

Contrary to our hypothesis, extraversion did not impact attention. Previously, individuals higher in extraversion have been shown to exhibit a smaller attentional blink^25^ suggestive of increased processing capacity. Moreover, extraversion has also been linked to performance in a change detection task, with individuals higher in extraversion showing improved accuracy^28,68^. However, extraversion has also been shown to be associated with slower reaction times during a change detection task, as well as improved accuracy, indicative of a speed-accuracy trade-off^68^. An important consideration when discussing findings from cognitive tasks is the characteristics of the task employed. The current study adopted a visual search task that requires less processing effort compared to the change detection task adopted in previous studies^28,68^ and may not have been sufficient to reveal any influence of extraversion. Nonetheless, the current finding furthers our understanding regarding the influence of extraversion on visual attention, demonstrating that extraversion does not impact reaction time in a visual search task involving the identification of a neutral target embedded within a real-world scene.

Neither extraversion, cognitive reappraisal, nor expressive suppression were shown to influence how accurately individuals identified the target in the visual search task. In contrast, individuals with higher levels of extraversion have previously displayed improved accuracy in a change detection flicker task compared to individuals with lower levels of extraversion^28,68^. One reason for the lack of any accuracy-related effects in the current study may be due to differences in the tasks adopted by researchers. For instance, it has been shown that in studies investigating emotional processing, task difficulty can influence the effects of emotional conditions on behavioural performance as well as neural activation in cognitive control and ER-associated brain regions. Specifically, in an n-back working memory task, more errors were made when words were negatively-valenced and the task condition was difficult. Further, corresponding neural activation measured with functional near-infrared spectroscopy and the late positive potential also revealed an interaction between valence and task difficulty in the PFC^69^. Interactions between task difficulty and emotional conditions have also been observed during the multi-source interference task. Here, both threat and reward distractors impacted reaction time of responding during incongruent trials (difficult condition) but not during no distractor trials (easy condition). Additionally, threat distractors decreased the neural response in cognitive control brain regions during incongruent trials whereas they increased the same neural response during congruent trials^70^. Future research is needed to (1) investigate whether affective individual difference traits such as extraversion and ER impact the effect of emotional stimuli on visual search, and (2) seek to clarify whether any such influences are impacted by task difficulty.

The current results also showed that extraversion, cognitive reappraisal, and expressive suppression did not interact with stimuli valence to influence visual search performance. These findings are surprising given that these traits are linked to improvements in affective reactivity with extraverts and individuals who habitually adopt ER strategies more successful in regulating and/or inhibiting emotional information. Moreover, adopting a psychometric approach, studies have shown that extraversion is positively related with attention to positive information and negatively correlated with attention to negative stimuli^32^. However, findings within the literature are not consistent with Lou et al.^33^ demonstrating that extraversion had no influence upon attentional biases to emotional stimuli in a modified oddball task. For ER, it has been shown that individuals who more frequently adopt cognitive reappraisal are quicker to disengage from sad faces compared to positive faces in a cued emotional conflict task^71^. Moreover, healthy individuals and MDD patients, who suffer from impairments in the control of attention and emotion, show a relationship between their performance in an emotional Stroop task (when negative stimuli were presented as distractors) and ER task completion^72^. One explanation for the lack of an interaction between stimuli valence and extraversion/ER in the current study could be that the task did not require sufficient attentional resources to reveal such effects (despite the task being able to identify main effects of stimuli valence and ER on visual search performance). For instance, task difficulty has been shown to influence the impact of emotional stimuli on working memory performance as well as performance during the multi-source interference task^69,70^. Therefore, future studies that adopt more challenging visual search paradigms may help to elucidate if affective traits interact with emotional valence to guide the allocation of attention. For instance, an extension of the current visual search task could vary the physical characteristics of real-world scenes and search targets by altering their luminance, contrast, or entropy, or by incorporating a secondary task to increase competition for cognitive resources. Such studies will help to develop our understanding of the individual differences involved in our ability to deploy attention within emotional situations.

Whilst the design of the current study provides several advantages, including the use of more naturalistic real-world stimuli to investigate visual search, the investigation of habitual ER strategy use rather than instructed ER, and the inclusion of both positive and negative conditions^73^, several limitations warrant consideration. Firstly, whilst the stimuli used in the current study were rigorously matched in their levels of luminance, contrast, and entropy, characteristics that have been shown to influence visual attention^74–76^, the stimuli sets differed in their levels of arousal. When stimuli are chosen based on valence it is common for the stimuli to also differ in their levels of arousal^77^. Therefore, it remains possible that the influences of stimuli valence on visual search reported in the current study may be due to variations in arousal rather than valence^78,79^. However, other studies have simultaneously investigated the influences of valence and arousal suggesting that valence (but not arousal) is the dominant influence on visual attention^80,81^. Future work is needed to investigate the independent influences of valence and arousal on visual search within real-world scenes. Secondly, we conducted a power analysis to ensure a suitable sample size and the study incorporated a large number of trials, yet additional work would benefit from using larger sample sizes to build upon the initial findings reported in the current study. This may be most beneficial when investigating individual differences at the facet level (e.g., extraversion) or when investigating interaction effects between traits and other variables associated with visual attention.

The novel observation that habitual use of a specific ER strategy, expressive suppression, influences the ability to correctly identify a neutral target within emotional real-world scenes may have implications for clinical research. Attentional training techniques have been shown to be successful as a neurobehavioral treatment of emotional disorders^82–84^. Moreover, ER therapy has shown clinical improvements for individuals suffering from anxiety and depression^85–87^. Treatments and therapies that simultaneously target changes in the allocation of attention *and* increased habitual use of effective ER strategies may provide more efficacious interventions for individuals suffering from affective disorders.

In conclusion, using a visual search task encompassing real-world stimuli of varying emotional valence, we demonstrate that positive and negative stimuli influence visual attention. Accuracy to correctly identify targets was reduced when the stimuli was positive compared to when the stimuli were neutral. In contrast, response time was reduced when the stimuli were neutral compared to negative. Moreover, we also show that individuals reporting more frequent habitual use of expressive suppression, were quicker at identifying search targets regardless of the emotional valence of the stimuli. These findings add to our knowledge regarding individual differences and their ability to impact the successful allocation of attention.

## Method

### Participants

Previous research investigating the influences of stimuli valence on visual attention using the visual search task adopted in the current study produced effect sizes of 0.21 and 0.35^16^. As such, we used an effect size of 0.25 to calculate our sample size. Using G*Power^88^, a sample size calculation for a regression model with four predictor variables, aiming to achieve statistical power of 0.95 with an alpha criterion of 0.05 and a medium effect size of 0.25, suggested that 45 participants were required. Participants were a convenience sample of 50 (40 female) staff and students from the University of Salford aged between 18 and 40 years (*M* = 25.30, *SD* = 6.50). Volunteers received a £10 inconvenience allowance and all participants provided informed consent. Ethical approval was obtained from the College of Health and Social Care Research Ethics Committee at the University of Salford. All methods were carried out in accordance with the relevant guidelines and regulations.

### Design

A mixed design was used with four independent variables. A within-participants categorical variable was the emotional valence of the stimuli used during the visual search task (positive, neutral, or negative). Between-participants continuous variables were levels of extraversion, cognitive reappraisal, and expressive suppression. Behavioural performance during the visual search task was assessed by recording accuracy (percentage correct) and response time (in seconds) to identify the search target.

### Materials

E-Prime (Psychology Software Tools, Inc.) was used to run the experiment and participants completed the study using a 60 Hz, ~ 43 cm LCD monitor. Images were selected from the Nencki Affective Picture System^89^ on the basis of their affective valence ratings and a total of 192 images were used for the visual search task; 64 positive, 64 neutral, and 64 negative images (identification numbers are provided in Supplementary Materials 1). All images were presented in colour and measured 1600 × 1200 pixels. The target in each visual search trial consisted of either a letter T or letter L and these were embedded within the image. The target letters were displayed at 0, 90, 180, or 270-degree orientations in Arial font size 12. To assess whether the stimuli sets differed according to valence a one-way analysis of variance (ANOVA) was conducted using SPSS. Mauchly’s test indicated that the assumption of sphericity had been violated therefore degrees of freedom were corrected using Huynh–Feldt estimates of sphericity (ε = 0.80). Analysis showed a significant main effect of valence, *F* (1.61, 101.20) = 2819.30, *MSE* = 0.19, *p* < 0.001, η_p_^2^ = 0.98. Planned contrasts revealed that positive stimuli (*M* = 7.70, *SD* = 0.34) had increased valence scores compared to neutral stimuli (*M* = 5.01, *SD* = 0.20), *F* (1, 63) = 3199.01, *MSE* = 0.15, *p* < 0.001, η_p_^2^ = 0.98. Neutral stimuli had higher valence scores than negative stimuli (*M* = 2.55, *SD* = 0.58), *F* (1, 63) = 1003.20, *MSE* = 0.39, *p* < 0.001, η_p_^2^ = 0.94. Stimuli sets were also assessed in respect of their luminance, contrast, entropy, and arousal. For luminance, Mauchly’s test indicated that the assumption of sphericity had been violated therefore degrees of freedom were corrected using Huynh–Feldt estimates of sphericity (ε = 0.90). The ANOVA showed that the three stimuli sets did not differ in their luminance (neutral *M* = 118.64, *SD* = 33.11, positive *M* = 120.55, *SD* = 30.47, negative *M* = 117.01, *SD* = 29.60), *F* (1.80, 113.51) = 0.202, *MSE* = 1102.96, *p* = 0.795, η_p_^2^ = 0.00. The three sets were also similar in contrast (neutral *M* = 65.10, *SD* = 19.92, positive *M* = 62.60, *SD* = 13.14, negative *M* = 62.82, *SD* = 12.22), *F* (2, 126) = 0.656, *MSE* = 187.47, *p* = 0.521, η_p_^2^ = 0.01, and entropy (neutral *M* = 7.50, *SD* = 0.36, positive *M* = 7.46, *SD* = 0.38, negative *M* = 7.52, *SD* = 0.38), *F* (2, 126) = 0.491, *MSE* = 0.145, *p* = 0.613, η_p_^2^ = 0.01. However, the stimuli sets did differ in their arousal ratings, *F* (1.64, 103.17) = 368.67, *MSE* = 0.45, *p* < 0.001, η_p_^2^ = 0.85. Planned contrasts revealed that positive stimuli (*M* = 3.81, *SD* = 0.96) had decreased arousal scores compared to neutral stimuli (*M* = 5.57, *SD* = 0.59), *F* (1, 63) = 221.34, *MSE* = 0.90, *p* < 0.001, η_p_^2^ = 0.78. Neutral stimuli had decreased arousal scores compared to negative stimuli (*M* = 6.71, *SD* = 0.63), *F* (1, 63) = 221.40, *MSE* = 0.37, *p* < 0.001, η_p_^2^ = 0.78.

The emotion regulation questionnaire (ERQ)^38^ was administered to assess habitual use of ER strategies. The 10-item self-report questionnaire includes questions in relation to how individuals regulate their emotions and provides scores on two ER strategies: cognitive reappraisal and expressive suppression. All items are reported on a 7-point scale from 1 to 7 (strongly disagree to strongly agree). Six questions assess cognitive reappraisal with a minimum score of 6 and a maximum score of 42. Four questions assess expressive suppression with a minimum score of 4 and a maximum score of 28. For both ER strategies low scores indicate lower levels of habitual strategy use whilst high scores indicate higher levels of habitual strategy use. The NEO Five-Factor Inventory-3 (NEO-FFI-3)^90^ was administered to measure levels of extraversion. The 60-item self-report scale includes questions relating to personality characteristics and traits along a 5-point Likert scale from 0 (strongly disagree) to 4 (strongly agree). Each trait is measured by 12 questions, the minimum score for each trait is 0 (indicating lower levels) and the maximum is 48 (indicating higher levels). In the current study the trait of interest was extraversion, but the full inventory was administered.

### Procedure

**Figure 3:**
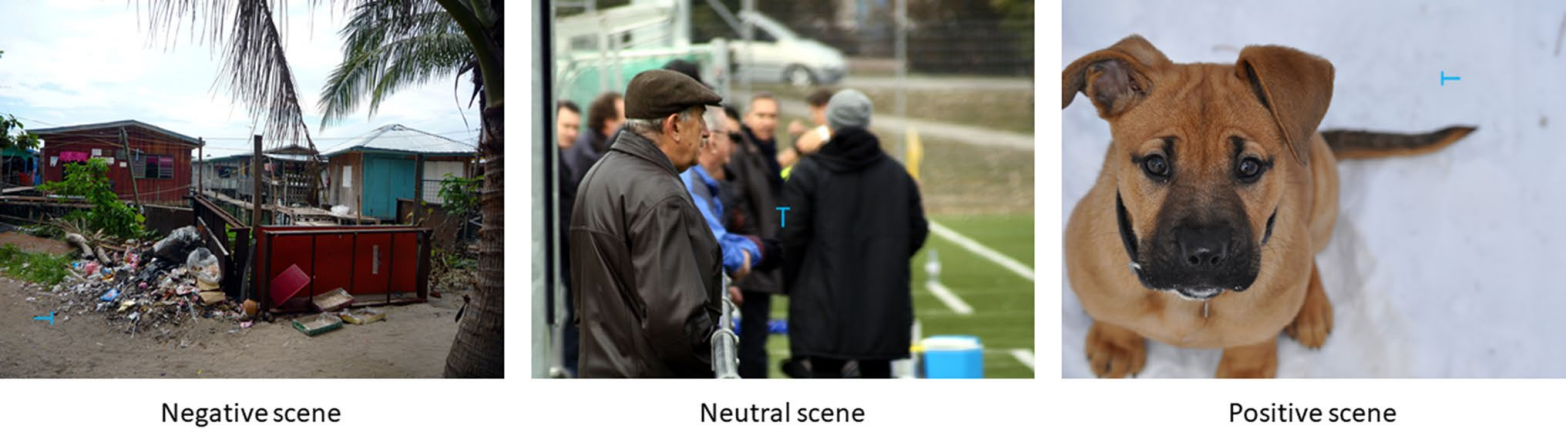
Representative examples of visual search stimuli. Targets were embedded within positive, neutral, and negative real-world scenes from the Nencki Affective Picture System^89^.

After providing written informed consent participants completed the ERQ and NEO-FFI-3. Participants were seated ~ 56 cm from the screen and a chin rest was used to reduce head movement and increase comfort. Participants were then given full instructions about the task. Following this, participants were shown onscreen instructions and were asked to press the spacebar when ready to begin. The visual search task was based on that detailed in Bendall et al.^16^ and adapted from Brockmole and Henderson^91^. Trials began with the presentation of a fixation cross for 1000 ms after which an image was presented with the letter T or L superimposed (Fig. 3). This image remained until the letter was identified with participants pressing the T or L key on the keyboard. Feedback was then provided before the next trial was initiated. In total participants completed 192 trials presented randomly. There were 64 trials for each valence condition (positive, negative, and neutral), 50% of these included the letter T and 50% showed the letter L. In these 32 trials the target letter was presented at a randomly selected central location (an area that extended 225 pixels to the left and right of fixation/centre and 171 pixels above and below fixation/centre) for 50% of the trials and presented at a random location outside of this central area for the remaining 50% of trials. An equal number of targets were presented at each angle of rotation in both the centre and periphery for each condition of stimuli valence.

## Supplementary Information

Supplementary Information.

## Data Availability

The datasets generated during and/or analysed during the current study are available from the corresponding author on reasonable request.
